# Predicting experiences of paranoia and auditory verbal hallucinations in daily life with ambulatory sensor data – A feasibility study

**DOI:** 10.1017/S0033291725000881

**Published:** 2025-04-11

**Authors:** Felix Strakeljahn, Tania M. Lincoln, Björn Schlier

**Affiliations:** 1 Clinical Psychology and Psychotherapy, Institute of Psychology, Faculty of Psychology and Movement Sciences, University of Hamburg, Hamburg, Germany; 2 Clinical Child and Adolescent Psychology and Psychotherapy, University of Wuppertal, Wuppertal, Germany

**Keywords:** EMA, JITAI, machine learning, psychosis, symptom prediction

## Abstract

**Background:**

Prediction models that can detect the onset of psychotic experiences are a key component of developing Just-In-Time Adaptive Interventions (JITAI). Building these models on passively collectable data could substantially reduce user burden. In this study, we developed prediction models to detect experiences of auditory verbal hallucinations (AVH) and paranoia using ambulatory sensor data and assessed their stability over 12 weeks.

**Methods:**

Fourteen individuals diagnosed with a schizophrenia-spectrum disorder participated in a 12-day Ecological Momentary Assessment (EMA) study. They wore ambulatory sensors measuring autonomic arousal (i.e., electrodermal activity, heart rate variability) and completed questionnaires assessing the intensity/distress of AVHs and paranoia once every hour. After 12 weeks, participants repeated the EMA for four days for a follow-up assessment. We calculated prediction models to detect AVHs, paranoia, and AVH-/paranoia-related distress using random forests within nested cross-validation. Calculated prediction models were applied to the follow-up data to assess the stability of prediction models.

**Results:**

Prediction models calculated with physiological data achieved high accuracy both for AVH (81%) and paranoia (69%–75%). Accuracy increased by providing models with baseline information about psychotic symptom levels (AVH: 86%; paranoia: 80%–85%). During the follow-up EMA accuracy dropped slightly throughout all models but remained high (73%–84%).

**Conclusions:**

Relying solely on physiological data to detect psychotic symptoms achieved substantial accuracy that remained sufficiently stable over 12 weeks. Experiences of AVHs can be predicted with higher accuracy and long-term stability than paranoia. The findings tentatively suggest that psychophysiology-based prediction models could be used to develop and enhance JITAIs for psychosis.

## Introduction

Envision a smartphone app that seamlessly integrates into the daily lives of people with psychosis. This app could serve as an ever-present guardian, either by anticipating the onset of psychotic symptoms to offer timely preventative interventions or by identifying significant symptom distress to deliver coping-oriented interventions. Such Just-In-Time Adaptive Interventions (JITAIs; Nahum-Shani, Hekler, & Spruijt-Metz, 2015; Spruijt-Metz & Nilsen, 2014) could provide timely, context-sensitive assistance and thus improve the treatment outcomes of state-of-the-art therapy for non-affective psychosis, which is often characterized by fluctuating or persisting (residual) symptoms (Harrison et al., 2001; Morgan et al., 2022, 2014; Peritogiannis, Gogou, & Samakouri, 2020).

In everyday life, such persisting symptoms could be experienced as repeated intervals of paranoia or hearing voices that predictably emerge and/or change in intensity over the day. JITAIs could be used to predict these distressing clinically relevant symptoms and then provide ambulatory interventions to deal with or even prevent symptom emergence (Juarascio, Parker, Lagacey, & Godfrey, 2018; Nahum-Shani et al., 2018).

To successfully develop JITAIs, one crucial component needed, next to effective ambulatory interventions, is an accurate prediction model for momentary symptoms (Nahum-Shani et al., 2018; Nahum-Shani, Hekler, & Spruijt-Metz, 2015). Compared to the ever-increasing field of research on psychosocial interventions for psychosis, however, research on these predictive models is still in its infancy and remains a significant challenge.

Momentary symptom experience prediction can rely either on actively collected data, typically multiple questionnaires per day collected via ecological momentary assessment (EMA), or on passively monitored parameters. In existing JITAI (pilot-)studies in psychosis, prediction models were based on EMA questionnaires and predefined thresholds to prompt interventions (Ben-Zeev et al., 2014, 2013). By contrast, models that utilize continuous passive monitoring of indicators, such as GPS location, physical activity, or sensor-based indicators of physiological parameters, e.g., heart rate variability or electrodermal activity, have not been tested for prediction models in patients with psychosis. Nonetheless, prediction models based on passively monitored parameters might be uniquely suited for JITAIs. Passive monitoring enables constant provision of real-time data, increasing the potential temporal resolution of prediction models (Kaiser et al., 2021). Further, passive monitoring is more feasible over prolonged periods of time due to minimized user burden caused by engagement with EMA questionnaires (Juarascio, Parker, Lagacey, & Godfrey, 2018; Nahum-Shani et al., 2018).

For psychotic symptoms, physiological indicators of change in autonomic nervous system activity (i.e., heart rate variability, electrodermal activity) are viable candidates for an accurate prediction model. First, the role of the autonomous nervous system and its complex interactions have been central both to the first conceptions of the influential vulnerability-stress model (Nuechterlein & Dawson, 1984) and to its more current iterations (Montaquila, Trachik, & Bedwell, 2015). Second, several EMA studies show that symptom experiences are preceded by changes in autonomic arousal (Cella et al., 2019; Kimhy et al., 2017; Schlier, Krkovic, Clamor, & Lincoln, 2019). Specifically, an EMA study found decreased heart rate variability to predict subsequent auditory verbal hallucinations in a clinical population (AVH; Kimhy et al., 2017). In an EMA study in a community sample with individuals with an increased likelihood of experiencing psychotic symptoms, decreased heart rate variability and increased electrodermal activity were shown during momentary experiences of paranoia, but no associations were found for hallucination spectrum experiences (Schlier, Krkovic, Clamor, & Lincoln, 2019). Finally, an EMA study that investigated changes in autonomic arousal between experiences of distressing vs. non-distressing psychotic symptoms (rather than between the mere presence vs. absence of psychotic symptoms) showed that electrodermal activity increases during distressing (vs. non-distressing) symptom experiences, but no changes in heart rate variability were found (Cella et al., 2019). Notably, research investigating the temporal associations between autonomic arousal and psychotic symptoms is limited. The methodological approaches used vary considerably across studies, and the results often conflict, complicating the evaluation of result consistency.

Despite these challenges, autonomic arousal might serve as a valuable predictor of psychotic symptoms particularly as a previous study from our lab indicates that it holds predictive value in detecting psychotic symptoms: In a proof-of-concept 24-hour EMA study on a population sample with attenuated levels of psychotic symptoms (Strakeljahn, Lincoln, Krkovic, & Schlier, 2024), we developed such prediction models for psychotic experiences using autonomic arousal (heart rate, heart rate variability, electrodermal activity) and physical activity parameters. We calculated random forest machine learning models, which yielded 65% sensitivity and 61% accuracy for predicting paranoia and 62% sensitivity (accuracy: 67%) for predicting hallucination-spectrum experiences in cross-validation. Adding self-reported lifetime psychotic symptom experiences as an additional predictor further increased sensitivity to 70% for paranoia (accuracy: 64%) and 68% for hallucination-spectrum experiences (accuracy: 74%). These results tentatively suggest that machine learning models using passive monitoring parameters can predict positive symptoms. Since these models were developed/tested in a healthy population, their accuracy in predicting AVHs and paranoia needs to be verified in patients with psychosis.

The present pre-registered study (osf.io/cvy8m) tests the accuracy with which machine learning models predict momentary experiences of paranoia and AVHs in the daily lives of patients diagnosed with a schizophrenia-spectrum disorder. First, we assessed the predictability of experiences of paranoia and AVH with machine learning models built with psychophysiological parameters (i.e., heart rate variability, electrodermal activity) and physical activity (to identify effort-based psychophysiological changes). Building upon the finding by Cella et al. (2019), we further investigated whether models that focused on predicting distressing symptom experiences rather than symptom experiences per se render themselves as an even more accurate prediction. Additionally, we tested whether including baseline levels of psychotic symptoms increases the accuracy of prediction models further. Finally, since in JITAIs, symptom prediction models need to function long-term, we investigated model longevity by testing the accuracy of machine learning models on follow-up data collected 12 weeks after the initial assessment period.

## Methods

### Design and procedure

Participants were recruited via (1) the outpatient clinic of the University of Hamburg, (2) leaflet advertisement at the University of Hamburg and nearby psychiatric facilities, and (3) via an internal list of people interested in participating in studies. Participants were screened via telephone or within the outpatient clinic for inclusion and exclusion criteria. Potentially eligible participants were invited for a baseline assessment in our laboratory. The baseline assessment aimed to verify the inclusion criteria and to assess baseline levels of positive symptoms of psychosis. All participants were interviewed with the Structured Clinical Interview for DSM-5 (SCID-5-CV; First & Williams, 2016) to determine if participants met the DSM-5 criteria for a schizophrenia spectrum disorder (i.e., schizophrenia, schizoaffective disorder, delusional disorder).

Participants filled out a demographical questionnaire, and the first author (FS) and a trained psychology master’s student conducted clinical interviews with them. Next, we showed participants how to equip the ambulatory sensors. Participants were also instructed on how to install and use the MovisensXS application (movisens GmbH, 2022) used for the EMA and how to start the ambulatory assessment on their smartphones. During the EMA period, notification prompts occurred between 9 am and 10:30 pm and were prompted on average every 60 min (± 10-minute random interval). At each notification prompt, participants answered questionnaires about their psychotic experiences and associated distress during the prior 20 min before the prompt. Participants were instructed to follow their normal daily routine during the assessment period. They were informed that they could participate in any light to normal activity, including sports, with the sensors and were requested to take off the sensors during intensive sports, while swimming, or while taking a shower or bath.

Participants were required to participate in the EMA for 12 days. We recommended them to complete the EMA in one 12-day interval, two six-day intervals, or three four-day intervals, but also accepted alternative EMA intervals provided that no break between the intervals exceeded three weeks.

Twelve weeks after the completion of the 12-day EMA period, participants were invited to a second interview assessment, which mirrored the baseline assessment, followed by another four-day EMA assessment.

For complete participation (i.e., 23 h of net study participation time including lab-visits for interviews at baseline and follow-up and answering times for all EMA-prompts), participants were compensated with 350 Euros (i.e., average hourly compensation of 15.22 Euros). The local ethics committee of the University of Hamburg approved the study (ID: 2022_016). All participants provided informed consent before participation. For further details regarding participant instructions to handle the sensors and a list of additional measures that are not central to the analyses of this study, see S1.

### Sample

To be eligible for this study, participants had to be at least 18 years old, be sufficiently proficient in German to answer the questionnaires, have a diagnosis of a schizophrenia spectrum disorder, have symptoms of paranoia and/or auditory verbal hallucinations that occur at least for several minutes on a daily basis and have persisted for at least three months, and be on stable medication for three months with no plans to change their medication within the next two months. Exclusion criteria were acute suicidality, a substance use or neurological disorder, a cardiovascular disease, or taking medication for cardiovascular problems.

The sample size rationale (pre-registration: osf.io/cvy8m) was based on the aim of building prediction models that theoretically could achieve a similar possible complexity to those in our previous study (Strakeljahn, Lincoln, Krkovic, & Schlier, 2024). This would allow us to better compare the prediction accuracy between the two studies as differences in prediction accuracy would not possibly be attributable to differences in the theoretical complexity achievable by the random forests. Assuming similar optimal hyperparameter configurations for prediction models across both studies, we estimated that for a 12-day EMA with 80% compliance we would need a minimum of 10 participants who complete the EMA, with at least five participants needing to experience paranoia and at least five needing to experience AVH. To achieve this aim of at least 10 viable participants in the face of potential attrition or critically low response rates in the EMA, we aimed to recruit 15 participants.

Of 19 participants who started the study, four were excluded for either not meeting the inclusion criteria (*n* = 2), deciding to discontinue following baseline assessment (*n* = 1), and or substantial difficulties in handling and maintaining the ambulatory sensors (*n* = 1). Another participant was excluded because of disclosing that they provided fake answers to the EMA questionnaires due to concerns about the assessment procedure. In total, 14 participants were included in the analyses, of which 13 completed the first EMA period whereas one provided only partial data after discontinuing the EMA. One participant missed a substantial number of prompts during the first EMA period and subsequently participated for two more days. Among these 14 participants, 13 experienced frequent paranoia and seven experienced regular AVHs. Eleven participants completed the follow-up (*n* = 11 with paranoia; *n* = 5 with AVHs). In the follow-up EMA, we asked two participants to take part in the EMA for six days because they showed low compliance rates in the first EMA period.

The mean participant age was 41.7 years (*SD* = 10.1), half of the sample identified as male and female, respectively, and 78.57% took antipsychotic medication, with 64.29% of participants fulfilling criteria for schizoaffective disorder and 35.71% for schizophrenia. The highest educational qualifications were either an intermediate secondary school certificate (42.86%) or a university entrance qualification (42.86%), with the remaining participants having completed a basic secondary school certificate (14.29%). The majority completed an apprenticeship (64.29%), while 21.43% reported having no formal professional qualifications, and 14.29% had a university degree. Employment status among participants showed that 42.86% were employed, with 7.14% working in a support facility, 28.57% classified as unable to work, and 21.43% were unemployed.

### Materials

#### Assessment of psychotic experiences

*Baseline assessment.* To assess baseline levels of paranoia and AVHs, we used the Psychotic Symptom Rating Scale (PSYRATS; Haddock, McCarron, Tarrier, & Faragher, 1999). The PSYRATS is a clinical interview that assesses the intensity and severity of experiences of delusions and auditory verbal hallucinations over the last week on a five-point rating scale (0–4). The delusion subscale of the PSYRATS consists of six items and the auditory verbal hallucinations subscale consists of 11 items. Mean scores were calculated for both subscales.

#### Daily life assessments

To assess experiences of paranoia, we used the five-item state version of the Paranoia Checklist (PCL-5; Freeman et al., 2005; Schlier, Moritz, & Lincoln, 2016). On an 11-point Likert scale (0 = *not at all* to 10 = *very much*), participants needed to indicate to what degree each item (e.g., “I need to be on my guard against others”) applied to them during the last 20 min (intensity scale). After each item, participants were further asked how much distress they were currently experiencing with the respective symptom (0 = *not at all* to 10 = *very much*). A mean score for each scale was calculated. To differentiate paranoia experiences from non-paranoia experiences, we followed the analytical strategy in our previous study (Strakeljahn, Lincoln, Krkovic, & Schlier, 2024) and calculated a 95%-reliable change index for the PCL-5 intensity and distress scale based on the within-subject Cronbach’s alpha and the variance from this sample. If scores were reliably different from the lower boundary of the mean score (0), they were marked as paranoia experiences. Based on the calculations of the 95% reliable change, we labeled assessments with PCL-5 intensity scores ≥ 1.90 as momentary paranoia events and with scores < 1.90 as events without paranoia. For the PCL-5 distress scale, we calculated a reliable change index as well (RCI = 1.81). To distinguish distressing experiences of paranoia from non-distressing experiences, we labeled assessments in which the PCL-5 intensity score was ≥ 1.90 and, at the same time, the PCL-5 distress score ≥ 1.81 as distressing paranoia events and all other instances as events without distressing paranoia.

To assess experiences of AVHs, we used the three items of the subscale auditory hallucinations from the Continuum of Auditory Hallucinations – State Assessment (CAHSA; Schlier, Hennig, & Lincoln, 2017). On an 11-point Likert scale (0 = *not at all* to 10 = *very much*), participants needed to indicate to what degree each item (e.g., “I heard something other people cannot hear”) applied to them during the last 20 min (intensity scale). After each item, participants were further asked how much distress they currently experience with the respective symptom (0 = *not at all* to 10 = *very much*). A mean score of each scale was calculated. To differentiate between assessments without AVHs and with AVHs, we calculated a reliable change index as described above. Assessments with CAHSA intensity scale scores ≥ 2.39 were labeled as momentary AVH events and scores < 2.39 as events without AVH. Additionally, assessments with CAHSA intensity scale scores ≥ 2.39 and, at the same time, distress scale scores ≥ 2.00 were labeled as distressing AVH events, and all other instances as events without distressing experiences of AVH.

#### Assessment of physiological predictors

Electrodermal activity was assessed with the EDAMove4 (movisens GmbH), an ambulatory electrodermal activity sensor that participants wore attached to a wristband on the wrist of their non-dominant hand. The wristband was equipped with two cables, to which single-use electrodes can be attached. The electrodes were placed on the outer palm. Heart rate variability, heart rate, and physical activity were assessed using an ambulatory electrocardiogram, the ECGMove4 (movisens GmbH), which participants wore via a chest belt at the level of the sternum. The raw sensor data was analyzed and automatically corrected for potential artifacts with DataAnalyzer (movisens GmbH, 2023). Using DataAnalyzer, we calculated a multitude of different variants of heart rate variability, electrodermal activity, and physical activity (see Supplementary Table S1 for the full list of physiological variables used). We used the physiological data in one-minute intervals from the 20-minute period prior to each EMA assessment. Individual one-minute heart rate and heart rate variability assessments deemed invalid by the DataAnalyzer were marked as missing. Additionally, we marked heart rate assessments as missing when they exceeded the maximal age-predicted heart rate for the respective participant according to the formula 208−0.7×age (Tanaka, Monahan, & Seals, 2001).

### Strategy of data analysis

All analyses were conducted in R 4.3.2 and Python 3.8.6. For the machine learning procedure, we used the package scikit-learn (Pedregosa et al., 2011).

Missings during the EMA were removed from the dataset of the first EMA period (214 assessments participants actively dismissed, 630 assessments ignored, 22 assessments incompletely filled out) and of the follow-up EMA period (91 assessments participants actively dismissed, 249 assessments ignored, 5 assessments incompletely filled out). In total, 65.29% of EMA prompts were answered and filled out completely in the first EMA period and 60.93% in the follow-up EMA period.

Missing data in physiological variables were imputed via k-Nearest Neighbors single imputation using the non-weighted mean of the three nearest neighbors. For a detailed list of missing data for each variable, see Supplementary Table S2.

Hyperparameter tuning and evaluation of the prediction models were conducted via nested cross-validation (20 outer folds and 20 inner folds). During nested cross-validation, we class-balanced each outer fold training dataset via random undersampling. Table 1 depicts the hyperparameter space used. During the hyperparameter tuning, we randomly tested 600 possible hyperparameter configurations (random search).

**Table 1.** Configuration of the hyperparameter space used for hyperparameter tuning

Hyperparameter	Possible values
Number of trees	250
Max. depth	2–50 in steps of two
Max. features per split	5%–80% in steps of five percent
Min. samples per leaf	2–30 in steps of two

To evaluate whether prediction models remained accurate over a 12-week period, we retrained the prediction model on the whole dataset of the first assessment period and applied the prediction model to the 12-week follow-up data. Again, the training dataset was class-balanced via random undersampling. Due to the fact that the nested cross-validation in the analysis before yielded different optimal hyperparameter configurations per outer fold, we decided to train the prediction model on the optimal hyperparameter configuration of each outer fold. Consequently, 20 prediction models with the optimal hyperparameter configuration of the respective outer fold were calculated, and we averaged the results. For our analyses, we focused on the following metrics to interpret the model quality: sensitivity (true positives/[true positives + false negatives]), specificity (true negatives/[true negatives + false positives]), positive predictive value (true positives/[true positives + false positives]), negative predictive value (true negatives/[true negatives + false negatives]), accuracy ([true positives + true negatives]/[true positives + true negatives + false positives + false negatives]), balanced accuracy ([sensitivity + specificity]/2).

## Results

### Descriptive data

Participants included in the paranoia prediction sample showed an average PSYRATS-delusion score of 2.5 (*SD* = 0.5, range = 1.3–3.2) during the baseline assessment (follow-up assessment: *M* = 2.1, *SD* = 0.6, range = 0.8–3.0). Participants included in the AVH prediction sample showed an average PSYRATS-AVH score of 2.4 (*SD* = 0.6, range = 1.2–3.2) during the baseline assessment (follow-up assessment *M* = 2.4, *SD* = 0.6, range = 1.7–2.8).

Of the 1526 completed assessments in the paranoia prediction sample 37.16% of assessments were classified as symptom experiences (567 assessments). Of the 567 experiences of paranoia, 84.48% were classified as distressing experiences of paranoia (479 assessments). For the AVH prediction sample (776 assessments completed), 34.41% of assessments were classified as experiences of AVH (267 assessments), of which 88.01% were classified as distressing experiences of AVH (235 assessments). The follow-up dataset for paranoia contained 538 EMA assessments of which 36.99% were classified as experiences of paranoia (199 assessments). Of the 199 assessments, 95.98% were classified as distressing experiences of paranoia (191 assessments). For AVH, the follow-up dataset contained 220 EMA assessments, of which 37.27% were classified as experiences of AVH (82 assessments). Of the 82 assessments, 92.68% were classified as distressing experiences of AVH (76 assessments).

### Nested cross-validation results

The nested cross-validation results for each prediction model are depicted in Table 2. The model to predict the occurrence of AVH in daily life yielded a sensitivity of 82% to detect AVH experiences and an overall accuracy of 81%. Predicting distressing experiences of AVH yielded a sensitivity of 84% (accuracy = 81%). Sensitivity for predicting paranoia intensity was 76% (accuracy = 75%) and for distressing experiences of paranoia 75% (accuracy = 69%).

**Table 2.** Nested cross-validation results for each prediction model

Model	Sensitivity	PPV	Specificity	NPV	BAC	Accuracy
AVH – intensity						
Physio	82%	69%	81%	90%	82%	81%
Physio+PSYRATS	90%	75%	84%	94%	87%	86%
AVH – distress						
Physio	84%	65%	80%	92%	82%	81%
Physio+PSYRATS	90%	71%	84%	95%	87%	86%
Paranoia – intensity						
Physio	76%	64%	75%	84%	76%	75%
Physio+PSYRATS	87%	76%	84%	92%	86%	85%
Paranoia – distress						
Physio	75%	50%	66%	85%	71%	69%
Physio+PSYRATS	87%	64%	77%	93%	82%	80%

*Note:* Physio, model based on only physiological data; Physio+PSYRATS, model based on physiological data and PSYRATS scores; PPV, positive predictive value; NPV, negative predictive value; BAC, balanced accuracy.

When adding the baseline diagnostic information about psychotic symptom levels (PSYRATS), descriptively the sensitivity increased further for both AVH intensity (90%) and distress (90%) and paranoia intensity (87%) and distress (87%).

### Temporal stability of prediction models

The follow-up results for each prediction model are depicted in Table 3. The prediction models achieved a sensitivity of 79% both to detect the occurrence of AVHs and distressing AVHs, descriptively resulting in decreases compared to the first EMA period by 3%–5% for the models using physiological parameters only. These decreases in sensitivity (18%–22%) were even more pronounced in the prediction models that included baseline information about psychotic symptom levels, with a sensitivity of 72% for the intensity AVH model and 68% for the distress AVH model.

**Table 3.** Accuracy of prediction models at follow-up after 12 weeks

Model	Sensitivity	PPV	Specificity	NPV	BAC	Accuracy
AVH – intensity						
Physio	79%	63%	73%	85%	76%	75%
Physio+PSYRATS	72%	77%	87%	84%	80%	82%
AVH – distress						
Physio	79%	63%	75%	87%	77%	76%
Physio+PSYRATS	68%	83%	93%	85%	81%	84%
Paranoia – intensity						
Physio	69%	67%	80%	81%	75%	76%
Physio+PSYRATS	42%	88%	97%	74%	70%	77%
Paranoia – distress						
Physio	72%	61%	74%	83%	73%	73%
Physio+PSYRATS	45%	77%	93%	75%	69%	76%

*Note:* Physio, model based on only physiological data; Physio+PSYRATS, model based on physiological data and PSYRATS scores; PPV, positive predictive value; NPV, negative predictive value; BAC, balanced accuracy.

Descriptively, the sensitivity for predicting (distressing) experiences of paranoia using physiological parameters was reduced by 3%–7% at the 12-week follow-up. Similar to AVH, the sensitivity of prediction models that added baseline psychotic symptom levels dropped markedly for both paranoia intensity (42%) and distress (45%).

### Exploratory analyses

We explored whether an alternative cut-off used for model calculation would affect prediction model accuracy. For this purpose, we focused on AVHs. Based on the premise that AVHs are either experienced or not, we calculated a prediction model to detect the occurrence of AVHs (AVH intensity scale) by labeling all assessments above the low point of the scale (> 0) as AVHs being present. The calculation of this prediction model followed the same methodology as the other prediction models in this study.

This prediction model to detect the occurrence of AVHs (AVH classified as prevalent in 46% of the assessments) using only physiological variables yielded a sensitivity of 75% (positive predictive value = 80%), a specificity of 84% (negative predictive value = 80%), and an accuracy of 80%. Applying this prediction model to the follow-up EMA, we saw a sensitivity of 80% (positive predictive value = 88%), a specificity of 85% (negative predictive value = 76%), and an accuracy of 82%.

## Discussion

This study tested whether psychophysiological indicators of changes in the autonomic nervous system’s activity can be utilized to predict the onset of AVHs and paranoia in people with psychosis. Using only psychophysiological data, the prediction models achieved up to 84% sensitivity for AVHs and up to 76% sensitivity for paranoia. The sensitivity (and accuracy) further increased by 6%–12% (and 5%–11%) when diagnostic information about symptom severity was added. In sum, all models achieved high sensitivity and accuracy in detecting the onset of (distressing) symptom experiences, with peak sensitivity of 90% for AVHs and 87% for paranoia.

Compared to our previous proof-of-concept study in a population sample (Strakeljahn, Lincoln, Krkovic, & Schlier, 2024), the present symptom-state prediction models in patients with psychosis yielded better results: Symptom prediction models using physiological predictors alone showed 11%–20% higher sensitivity (14% higher accuracy), and models additionally using baseline symptom levels showed 17%–22% higher sensitivity (12%–21% higher accuracy). Possibly, patients with psychosis show more pronounced symptoms and/or more prominent fluctuations in psychophysiological parameters, allowing for a better distinction between symptom and no-symptom states. The assumption of more fluctuation in psychophysiological arousal is in line with EMA studies that consistently found people with psychosis to self-report increased emotional reactivity to stress (Myin-Germeys & van Os, 2007) and affective instability (Nowak, Krkovic, Kammerer, & Lincoln, 2022). However, this study had more EMA assessments per person (*M* = 116 EMA assessments) than its predecessor (*M* = 33; Strakeljahn, Lincoln, Krkovic, & Schlier, 2024), which itself could have allowed for a more precise estimation of the individual relationships between changes in autonomic arousal and state symptoms. A future direct comparison of clinical and subclinical groups using the same assessment schedule could elucidate if and to what degree improved accuracy in patients was an artifact of more data per person. Additionally, the distribution of assessments with symptom experiences and non-symptom experiences differed between the two studies. While in this study 37.16% of assessments were classified as experiences of paranoia (AVH = 34.41%) participants in the previous study (Strakeljahn, Lincoln, Krkovic, & Schlier, 2024) reported fewer paranoia experiences (20.30%; hallucination spectrum experiences = 27.04%). These variations in the distribution of symptom experiences between clinical and subclinical populations might also have an impact on model accuracy.

In this study, participants rated over 80% of all AVH and paranoia experiences as distressing. Prediction models’ sensitivity remained stable to slightly increased when predicting distressing AVHs. This is particularly interesting because many recent innovations in psychotherapeutic treatments for voice hearing, e.g., AVATAR therapy (Craig et al., 2018; Ward et al., 2020) or Relating Therapy (Hayward et al., 2017; Lincoln et al., 2024), focus on treating AVH distress rather than AVH-presence to improve patients’ functioning and quality of life. With consistently high sensitivity for AVH distress, our prediction models could prove to be an ideal fit for future hallucination-focused, relational JITAIs.

Comparing our prediction models for psychotic symptoms with the accuracy of machine learning models using psychophysiological parameters to predict psychological states such as stress, the accuracy we obtained is well within the range of the overall literature on predicting psychological states: A recent review of studies predicting stress via passively monitored psychophysiological parameters reported 65%–98% accuracy across all studies (Vos, Trinh, Sarnyai, & Rahimi Azghadi, 2023), mirroring the 69%–86% accuracy range across our models. Since various psychological states can be predicted via autonomic arousal, the question remains whether our models specifically detect psychotic symptoms or overall psychopathological distress. Since our prediction models predicted the presence of psychotic experiences in general and distressing psychotic experiences in specific with comparable accuracy, one might argue that our prediction models do not merely track participants’ distress. Since the distressing symptom experiences constituted the vast majority of all symptom experiences, however, differentiation and specificity of these states still need further exploration in future studies with larger, more diverse samples. If prediction models using psychophysiological indicators turn out not to be specific to certain symptoms, future implementation strategies need to account for that. For example, JITAIs could prompt a selection of self-help interventions or guide patients towards the most fitting intervention by starting with a short questionnaire to pinpoint what symptoms are emerging.

To our knowledge, this is the first study of its kind to assess the long-term stability of its prediction models (for psychotic symptoms or pathological states in general). The evaluation of our prediction models at the 12-week follow-up showed that, despite a decrease in accuracy across all prediction models, all models retained sufficient accuracy (73%–84%). The AVH prediction models also showed consistently high sensitivity (68%–79%) and accuracy (75%–84%) at follow-up. For paranoia, sensitivity and accuracy of the physiological prediction models remained relatively stable at follow-up, whereas paranoia prediction models utilizing diagnostic information on current psychotic symptom levels showed dramatically lowered sensitivity (42%–45%) to or below chance levels, whereas overall accuracy remained high. In terms of implementing these prediction models in a JITAI for paranoia, this lack of stability poses a problem. A prediction model like this utilized for JITAI would shift towards missing more relevant paranoia episodes, potentially frustrating users or leading to disengagement.

Taking all these results together and in line with our previous study (Strakeljahn, Lincoln, Krkovic, & Schlier, 2024), the prediction of AVHs/distressing AVHs yielded a higher accuracy across all models and assessment periods than the paranoia prediction models. Possibly, autonomic arousal as a set of predictors is best suited to predict episodic-experiential pathological states (such as the impression of hearing a voice), whereas cognitive-reflexive states (such as paranoid thinking) need further predictors. Beyond the utilization of psychophysiological parameters, several EMA studies identified other candidates for passively monitored parameters that might be suited for the prediction of psychotic experiences. For example, speech detection and communication habits as monitored via smartphone constitute promising additions for prediction models, as the length of outgoing phone calls and the time spent in environments where human speech was detected correlated with EMA-reported paranoia (Buck et al., 2019). Further, dynamic vulnerability factors (e.g., poor sleep) associated with increases in experiencing psychotic symptoms (Reeve, Emsley, Sheaves, & Freeman, 2018) could be added into symptom prediction models to guide machine learning models as a quasi-moderating factor. Of importance, speech detection, physical activity, and sleep have already been successfully utilized to predict weekly levels of psychotic symptoms (Wang et al., 2017). Potentially, the combination of psychophysiological parameters and these mobile sensing parameters could increase the prediction accuracy of machine learning models for paranoia and further strengthen their long-term stability.

We differentiated between the presence and absence of psychotic symptoms by using a cut-off based on a reliable change index to dichotomize outcomes. Other options for operationalizing psychotic symptom presence exist, such as a fixed-value cut-off, e.g., the scale midpoint (Cella et al., 2019) or all values above the lowest point (Schlier, Winkler, Jaya, & Lincoln, 2018). To explore how a different cut-off might influence prediction accuracy, we tested labeling AVHs as present when participants’ AVH score was above the scale’s lowest point (> 0). This analysis showed that a physiological prediction model achieved comparable accuracy (80%) as the AVH prediction model using the reliable change index (81%). Nonetheless, some metrics showed favorable results for the prediction model using the low point of the scale with higher positive predictive value and specificity but lower sensitivity and negative predictive value. Further, this prediction model exhibited greater stability with an accuracy of 82% at follow-up compared to 75% with the reliable change index model. Consequently, using different cut-off points for prediction models may offer diverse advantages/disadvantages regarding prediction model accuracy. In this study, a low cut-off point led to higher precision in terms of positive predictive value. As a result, a JITAI that would prompt interventions based on this prediction model would produce fewer false alarms than a prediction model using the reliable change index as a cut-off but with the trade-off of missing more AVH episodes.

The choice of a suitable cut-off for JITAI could ultimately be a complex, even completely personalized, decision depending on the goal of the JITAI for the respective target group or person. JITAIs aimed at preventing symptom emergence might benefit from utilizing lower cut-off points to detect small differences in symptom changes to identify early symptom formation. Conversely, for individuals who experience symptoms, such as voices, almost constantly, the mere detection of any symptoms would be an unfeasible trigger to prompt an intervention. As previously discussed (Strakeljahn, Lincoln, Krkovic, & Schlier, 2024), for this group, cut-offs that correspond to less frequent clinically meaningful events, such as substantial voice distress, constitute a more useful cut-off.

We chose to use the reliable change index in this study to mirror the analytic choices of our previous study (Strakeljahn, Lincoln, Krkovic, & Schlier, 2024), to increase comparability between the prediction models calculated, and because this approach constitutes a diagnostically elaborate way to distinguish symptom and non-symptom experiences. We do not know if participants would have wanted interventions to be offered when exceeding this particular threshold. To address this issue and possibly establish personalized cut-offs, future studies could ask individuals throughout the EMA whether they would have desired support at each assessment interval. This feedback could be used to evaluate whether universal, group-optimized, or individually tailored cut-offs yield greater clinical usefulness.

To conclude, future studies could systematically examine variations in predictive accuracy across different cut-offs to determine which are most useful for JITAIs and to identify cut-offs tailored to specific JITAI objectives.

Some limitations must be considered: In this study, we descriptively compared the accuracy of prediction models with each other. However, various factors might contribute to changes in prediction accuracy between models. While one reason could be true variations in model performance, another important factor is the specific training and test datasets used for prediction model calculation. To mitigate variations in predictive accuracy introduced by the train/test split between prediction models, we utilized nested cross-validation (Singh et al., 2021). Nonetheless, changes in accuracy between prediction models could still be attributed to the train/test datasets. Additional replication studies are necessary to investigate whether specific symptoms (paranoia or AVH or their distress) show higher accuracy than others.

Moreover, similar to multiple other EMA studies with repeated measures that developed machine learning prediction models for various psychological states (Dalmeida & Masala, 2021; Velmovitsky et al., 2022; Wu et al., 2015), we used random forests. However, as pointed out in a recent review of longitudinal data analysis for random forests, random forests assume that the data points used to calculate the prediction models are independent from one another (Hu & Szymczak, 2023). As we could not explicitly capture the data’s hierarchical structure in random forests, we tried to provide information about its structure implicitly. Specifically, we provided prediction models with information about baseline levels of psychotic symptoms. While this resulted in increased predictive accuracy for the initial EMA assessment, accuracy during follow-up diverged: AVH prediction models utilizing baseline information remained sensitive and accurate, whereas the corresponding paranoia prediction models dramatically lost their sensitivity. Multiple explanations could account for these results. First, the prediction models might have used the PSYRATS scores to determine the number of expected psychotic experiences when there were no reliable indicators between assessments. By the follow-up, the PSYRATS delusion scores had decreased, potentially suggesting to the prediction model that participants experienced less paranoia during the follow-up EMA period. However, during the follow-up EMA period, the percentage of paranoia experiences of all EMA assessments remained numerically stable, and the frequency of distressing experiences of paranoia even numerically increased. In line with this explanation, the paranoia prediction models that solely used physiological data remained comparatively stable. Second, implicitly providing prediction models with information about the hierarchical structure of the data might have led to overfitting in our small sample, resulting in poorer prediction performance at follow-up. Further, future developments of prediction models built with longitudinal data may benefit from utilizing recently developed extensions of the random forests algorithm, which are explicitly designed to be able to handle longitudinal data (Hu & Szymczak, 2023).

To conclude, this study demonstrated that predicting psychotic experiences using passively collectable variables is possible and that the prediction models remained relatively stable over several months. However, to ensure the suitability of the prediction models developed in this study for JITAI, issues such as long-term stability need to be tackled and the overall accuracy and precision of prediction models need to increase. Additionally, the specificity of prediction models for various psychological states (e.g., stress, negative affect, anxiety) remains to be determined. If prediction models are found to identify distress across several psychopathological states, additional passive-sensing predictors need to be identified to discriminate between these states. Furthermore, when prediction models’ accuracy, stability, and precision are further improved, they could then be implemented in a JITAI designed for individuals diagnosed with a schizophrenia-spectrum disorder.

## Supporting information

Strakeljahn et al. supplementary material Strakeljahn et al. supplementary material

## Supplementary material

The supplementary material for this article can be found at http://doi.org/10.1017/S0033291725000881.
