# Supplementary material for: Predicting experiences of paranoia and auditory verbal hallucinations in daily life with ambulatory sensor data – A feasibility study
Source: Psychol Med. 2025 Apr 11;55:e114. doi: 10.1017/S0033291725000881 (PMC12094635; doi:10.1017/S0033291725000881)
Supplement: Strakeljahn et al. supplementary material [file S0033291725000881sup001.docx]

Supplementary Material

Predicting experiences of paranoia and auditory verbal hallucinations in daily life with ambulatory sensor data – A feasibility study

Felix Strakeljahn^1^

Tania Lincoln^1^

Björn Schlier^2^

^1^Clinical Psychology and Psychotherapy, Institute of Psychology, Faculty of Psychology and Movement Sciences, University of Hamburg, Hamburg, Germany

^2^Clinical Child and Adolescent Psychology and Psychotherapy, Bergische Universität Wuppertal, Wuppertal, Germany

# Supplement 1

## Additional Information about the study’s procedure

The following measures were assessed over the course of the study but are not central to the current analyses of this study and, therefore, not reported: During the baseline and follow-up assessment, we additionally measured negative symptoms. During the EMA, at each assessment, we measured stress levels, negative affect, caffeine intake, smoking, intake of drugs, and whether people were physically active (e.g., did sports) in the last 20 minutes.

Participants were instructed in the handling of the sensors. Specifically, we showed participants how to equip the sensors independently and how to recharge them properly each night. For the EMA period we gave each participant a contingent of single-use electrodes for the electrodermal activity sensor and advised them to always replace the electrodes when they became loose or in contact with water. Participants were advised to equip the sensors in the morning, preferably after showering, wear them throughout the day, and unequip them before going to sleep. Additionally, participants received a short instruction manual to be able to reread how to equip and charge the sensors during the study. If participants faced difficulties with the sensors or the study in general, they were advised to contact the researchers for further help.

**Table S1**

*Physiological variables used for the calculation of prediction models*

| Physiological variables |
| --- |
| Heart Rate (Hr) |
| High Frequency (HrvHf) |
| Low Frequency (HrvLf) |
| HRV parameter Low to High Frequency Ratio LF/HF (HrvLfHf) |
| HRV parameter RMSSD (HrvRmssd) |
| HRV parameter pNN50 (HrvPnn50) |
| HRV parameter SDNN (HrvSdnn) |
| Mean Skin conductance level (EdaSclMean) |
| Number of SCR (EdaScrCount) |
| Mean of SCR Amplitudes (EdaScrAmplitudesMean) |
| Mean of SCR rise times (EdaScrRiseTimesMean) |
| Mean of SCR recovery times (EdaScrHalfRecoveryTimesMean) |
| Mean of SCR energy (EdaScrEnergiesMean) |
| Mean Arousal (EdaArousalMean) |
| Movement Acceleration (MovementAcceleration) |
| Step Count (StepCount) |
| Vertical Speed (VerticalSpeed) |

*Note.* The variables were calculated via the DataAnalyzer. The One-minute intervals of the 20-minute period prior to each assessment were used. For the exact calculation of the variables see the documentation of the DataAnalyzer (<https://docs.movisens.com/Algorithms/#sensor-data>).

**Table S2**

*Number of missing values per one-minute interval of each variable*

| Variable | Minutes prior to each assessment | | | | | | | | | | | | | | | | | | | |
| --- | --- | --- | --- | --- | --- | --- | --- | --- | --- | --- | --- | --- | --- | --- | --- | --- | --- | --- | --- | --- |
|  | 20 | 19 | 18 | 17 | 16 | 15 | 14 | 13 | 12 | 11 | 10 | 9 | 8 | 7 | 6 | 5 | 4 | 3 | 2 | 1 |
| Heart Rate (Hr) | 266 | 265 | 263 | 263 | 263 | 264 | 257 | 255 | 257 | 260 | 262 | 264 | 262 | 265 | 263 | 263 | 265 | 264 | 270 | 268 |
| High Frequency (HrvHf) | 327 | 327 | 317 | 315 | 316 | 317 | 319 | 310 | 314 | 323 | 323 | 318 | 312 | 308 | 319 | 316 | 314 | 321 | 323 | 323 |
| Low Frequency (HrvLf) | 327 | 327 | 317 | 315 | 316 | 317 | 319 | 310 | 314 | 323 | 323 | 318 | 312 | 308 | 319 | 316 | 314 | 321 | 323 | 323 |
| HRV parameter Low to High Frequency Ratio LF/HF (HrvLfHf) | 327 | 327 | 317 | 315 | 316 | 317 | 319 | 310 | 314 | 323 | 323 | 318 | 312 | 308 | 319 | 316 | 314 | 321 | 323 | 323 |
| HRV parameter RMSSD (HrvRmssd) | 327 | 327 | 317 | 315 | 316 | 317 | 319 | 310 | 314 | 323 | 323 | 318 | 312 | 308 | 319 | 316 | 314 | 321 | 323 | 323 |
| HRV parameter pNN50 (HrvPnn50) | 327 | 327 | 317 | 315 | 316 | 317 | 319 | 310 | 314 | 323 | 323 | 318 | 312 | 308 | 319 | 316 | 314 | 321 | 323 | 323 |
| HRV parameter SDNN (HrvSdnn) | 327 | 327 | 317 | 315 | 316 | 317 | 319 | 310 | 314 | 323 | 323 | 318 | 312 | 308 | 319 | 316 | 314 | 321 | 323 | 323 |
| Mean Skin conductance level (EdaSclMean) | 3 | 3 | 3 | 3 | 3 | 3 | 3 | 3 | 3 | 3 | 3 | 3 | 3 | 3 | 3 | 3 | 3 | 3 | 3 | 3 |
| Number of SCR (EdaScrCount) | 3 | 3 | 3 | 3 | 3 | 3 | 3 | 3 | 3 | 3 | 3 | 3 | 3 | 3 | 3 | 3 | 3 | 3 | 3 | 3 |
| Mean of SCR Amplitudes (EdaScrAmplitudesMean) | 3 | 3 | 3 | 3 | 3 | 3 | 3 | 3 | 3 | 3 | 3 | 3 | 3 | 3 | 3 | 3 | 3 | 3 | 3 | 3 |
| Mean of SCR rise times (EdaScrRiseTimesMean) | 3 | 3 | 3 | 3 | 3 | 3 | 3 | 3 | 3 | 3 | 3 | 3 | 3 | 3 | 3 | 3 | 3 | 3 | 3 | 3 |
| Mean of SCR recovery times (EdaScrHalfRecoveryTimesMean) | 3 | 3 | 3 | 3 | 3 | 3 | 3 | 3 | 3 | 3 | 3 | 3 | 3 | 3 | 3 | 3 | 3 | 3 | 3 | 3 |
| Mean of SCR energy (EdaScrEnergiesMean) | 3 | 3 | 3 | 3 | 3 | 3 | 3 | 3 | 3 | 3 | 3 | 3 | 3 | 3 | 3 | 3 | 3 | 3 | 3 | 3 |
| Mean Arousal (EdaArousalMean) | 3 | 3 | 3 | 3 | 3 | 3 | 3 | 3 | 3 | 3 | 3 | 3 | 3 | 3 | 3 | 3 | 3 | 3 | 3 | 3 |
| Movement Acceleration (MovementAcceleration) | 11 | 11 | 11 | 11 | 11 | 11 | 11 | 11 | 12 | 12 | 12 | 12 | 12 | 12 | 12 | 12 | 12 | 12 | 12 | 12 |
| Step Count (StepCount) | 11 | 11 | 11 | 11 | 11 | 11 | 11 | 11 | 12 | 12 | 12 | 12 | 12 | 12 | 12 | 12 | 12 | 12 | 12 | 12 |
| Vertical Speed (VerticalSpeed) | 11 | 11 | 11 | 11 | 11 | 11 | 11 | 11 | 12 | 12 | 12 | 12 | 12 | 12 | 12 | 12 | 12 | 12 | 12 | 12 |
| Participant ID | 0 | 0 | 0 | 0 | 0 | 0 | 0 | 0 | 0 | 0 | 0 | 0 | 0 | 0 | 0 | 0 | 0 | 0 | 0 | 0 |
| PSYRATS auditory hallucinations | 0 | 0 | 0 | 0 | 0 | 0 | 0 | 0 | 0 | 0 | 0 | 0 | 0 | 0 | 0 | 0 | 0 | 0 | 0 | 0 |
| PSYRATS delusions | 0 | 0 | 0 | 0 | 0 | 0 | 0 | 0 | 0 | 0 | 0 | 0 | 0 | 0 | 0 | 0 | 0 | 0 | 0 | 0 |

*Note*. PSYRATS = psychotic symptom rating scale; HRV = heart rate variability; SCR = skin conductance response.
